# Accurate Distinction of Pathogenic from Benign CNVs in Mental Retardation

**DOI:** 10.1371/journal.pcbi.1000752

**Published:** 2010-04-22

**Authors:** Jayne Y. Hehir-Kwa, Nienke Wieskamp, Caleb Webber, Rolph Pfundt, Han G. Brunner, Christian Gilissen, Bert B. A. de Vries, Chris P. Ponting, Joris A. Veltman

**Affiliations:** 1 Radboud University Nijmegen Medical Centre, Department of Human Genetics, Nijmegen, The Netherlands; 2 MRC Functional Genomics Unit, University of Oxford, Department of Physiology, Anatomy and Genetics, Oxford, United Kingdom; Uppsala University, Sweden

## Abstract

Copy number variants (CNVs) have recently been recognized as a common form of genomic variation in humans. Hundreds of CNVs can be detected in any individual genome using genomic microarrays or whole genome sequencing technology, but their phenotypic consequences are still poorly understood. Rare CNVs have been reported as a frequent cause of neurological disorders such as mental retardation (MR), schizophrenia and autism, prompting widespread implementation of CNV screening in diagnostics. In previous studies we have shown that, in contrast to benign CNVs, MR-associated CNVs are significantly enriched in genes whose mouse orthologues, when disrupted, result in a nervous system phenotype. In this study we developed and validated a novel computational method for differentiating between benign and MR-associated CNVs using structural and functional genomic features to annotate each CNV. In total 13 genomic features were included in the final version of a Naïve Bayesian Tree classifier, with LINE density and mouse knock-out phenotypes contributing most to the classifier's accuracy. After demonstrating that our method (called GECCO) perfectly classifies CNVs causing known MR-associated syndromes, we show that it achieves high accuracy (94%) and negative predictive value (99%) on a blinded test set of more than 1,200 CNVs from a large cohort of individuals with MR. These results indicate that this classification method will be of value for objectively prioritizing CNVs in clinical research and diagnostics.

## Author Summary

Rare copy number variants (CNVs) are a frequent cause of neurological disorders such as mental retardation (MR). However CNVs are also commonly identified in healthy individuals. It is therefore crucial for both diagnostic and research applications to be able to distinguish between disease-causing CNVs and “benign” CNVs occurring as normal genomic variation. Separating these two types can take advantage of significant differences in their genomic contents. For example, benign CNVs are enriched in repetitive sequences. By contrast, CNVs associated with MR tend to have high densities of functional elements, including genes whose mouse orthologues, when knocked-out, lead to specific nervous system abnormalities. We have developed a novel objective approach that is effective in distinguishing MR-associated CNVs from benign CNVs based on the presence of 13 genomic attributes. This method is able to achieve high accuracies in a cohort of CNVs known to cause MR and in a cohort of individuals with unexplained MR. The development of this technique promises to substantially improve the methodology for determining the pathogenicity of CNVs.

## Introduction

Improvements in microarray resolution and hybridization robustness have resulted in the widespread implementation of genomic microarray technologies in medical research and diagnostics. This technology is most effective in detecting genomic deletions and duplications larger than 1kb, known as copy number variants (CNVs). Genomic microarrays are commonly used to identify rare, but highly penetrant, and commonly single CNVs in patients suffering from neurological disorders such as autism [1]–[3], schizophrenia [4]–[6] and mental retardation (MR; also known as learning disability) [7]–[9]. However CNVs have also been recently recognized as a common form of genomic structural variation: high resolution microarrays and sequencing approaches are able to identify 600–900 CNVs in a single individual [10]–[14]. Current clinical interpretation therefore needs to contrast the frequencies of a CNV in affected *versus* unaffected individuals, as well as determining the inheritance of CNVs via parental analysis [15], [16]. The identification of a CNV that is (1) relatively large, (2) overlaps genes, (3) is rare, and (4) *de novo* in a patient provides a strong indicator of clinical significance, because this combination is extremely rare in the normal population owing to a low structural mutation rate outside of hypervariable ‘hot spot’ regions [10], [17], [18]. Increases in microarray resolution are revealing both a much higher rate of rare CNVs than previously thought [19] and an increasing number of genomic loci being reported that show variable inheritance and penetrance. Such examples have been reported for CNVs at 1q21.1 [20], [21], 15q13.3 [22], [23], and 16p13.11 [24], [25]. These loci demonstrate that there are limitations in considering CNVs as either benign when common and inherited, or causal when rare and *de novo*.

At present up to 5% of the human genome has been shown to vary in large scale copy number in numerous healthy controls [13], [26] and novel CNVs continue to be identified [27]. In Nguyen et al. (2008) [28] we reported a number of genomic features whose frequencies are significantly different in apparently benign CNV regions compared with the genome as a whole. In particular, CNV regions are enriched in repetitive sequences of near identical DNA known as segmental duplications [29] and are less prone to recombination. Furthermore, these CNV regions are characterized by tendencies to coincide with between-species break-points in synteny and to be prone to elevated nucleotide substitution rates, whilst their encoded proteins tend to exhibit elevated evolutionary rates. In a separate study we compared a large set of rare *de novo* CNVs associated with MR with CNVs identified in healthy control individuals. This study demonstrated that MR-associated CNVs are significantly enriched in genes whose mouse orthologues, when disrupted, result in abnormal axon or dopaminergic neuron morphologies, and in genes from neurodegenerative disease pathways [30]. Importantly, we showed that benign CNVs do not display such properties. Such observations can thus now be used to prioritize dosage-sensitive candidate genes for MR. Of relevance to this study is that these distinctions of MR-associated CNVs may be exploited to aid the development of an objective method for distinguishing disease-associated CNVs from benign CNVs that does not rely solely on allele inheritance and frequency in the normal population.

Although a large number of methods are available for the computational prioritization and classification of genomic data [31]–[34], none thus far has been developed specifically for CNV data. For this study we implement a Naïve-Bayesian Tree classifier (NBTree). This hybrid approach combines a decision tree with Naïve-Bayesian classifiers, and exploits the segmentation of decision trees and the accumulation of Naïve-Bayes evidence. There are four major advantages of decision-tree classifiers for assigning pathogenicity to CNVs. These classifiers are (*i*) fast and (*ii*) their results are easily comprehensible. They are (*iii*) very robust to irrelevant features and (*iv*) classification takes into account evidence from many attributes in arriving at a final prediction [35]. In this study our aim was to validate the use of an NBTree, based upon genomic features, to accurately separate disease-associated CNVs from benign CNVs.

## Results

We started by selecting genomic features **(** **Table 1** **)**, based on our previous observations [28], [30], as the basis attribute set for development of the classification procedure. In addition, we collected a large cohort of CNVs identified in healthy controls (termed “benign CNVs”) and a large set of CNVs associated with MR (termed “MR-associated CNVs”) [30]. These CNVs were used for training and testing the Naïve-Bayesian Tree classifier (NBTree). After optimization, the accuracy of the classifier was initially assessed by applying the classifier to a small independent set of CNVs known to be pathogenic (“Decipher known syndromes”). We subsequently applied the classifier to a third, much larger set of CNVs identified during routine MR microarray diagnostics, termed “MR diagnostics CNVs” (see **Figure S1** for study design). Finally, we studied two further sets of CNVs whose clinical significance is currently unknown. The first contained rare CNVs for which inheritance could not be determined (“candidate CNVs”). The second set contained rare, privately inherited CNVs.

**Table 1:** Genomic attributes investigated as potential classification features.

	Genomic Feature	Structural	Functional	Categorical	Continuous
**1**	Type (Gain/Loss)	*		*	
**2**	Length	*			*
**3**	# LINEs	*			*
**4**	LINE density	*			*
**5**	# SINEs^2^	*			*
**6**	SINE density	*			*
**7**	# Segmental Duplications	*			*
**8**	Segmental Duplication Density	*			*
**9**	# Genes^1^		*		*
**10**	Gene Density^1^		*		*
**11**	*d_S_* 3		*		*
**12**	*d_n_* 2 ^*,*^ 4		*		*
**13**	*d_n_*/*d_S_* 2		*		*
**14**	KEGG Pathway (hsa01510)		*	*	
**15**	MGI Phenotype (MP:0003631)		*	*	
**16**	Gene expression		*		*

Each feature is either categorical or a continuous numerical feature. Furthermore, each feature relates to either a structural genomic attribute or a functional genomic attribute.

*^1^For these features we counted the number of genes overlapping the CNV.*

*^2^These features did not contribute to the accuracy of the classifier and were removed from the final version.*

*^3^dS = Synonymous substitution rate.*

*^4^d_n_ = Non- synonymous substitution rate.*

### Development of the Classifier

We identified a total of 16 genomic features as suitable attributes for the classifier which could be divided into either: (1) structural features such as segmental duplication density, and (2) functional features, such as gene density **(** **Table 1** **)**. These genomic attributes were also considered to be either continuous or categorical features. To compensate for the dependencies of CNV length on the frequencies of features (e.g. LINE, SINE, segmental duplication and gene numbers) we also calculated the densities of LINEs, SINEs, segmental duplications and ENSEMBL gene models. A categorical feature was created to be set as ‘true’ when a CNV contains at least one gene whose mouse orthologue, when disrupted exhibits a mouse nervous system phenotype (and otherwise ‘false’). Previously we have shown that MR CNV genes are enriched in the KEGG neurodegenerative pathway (namely, hsa01510). This feature was also represented in the classifier, specifically as a categorical feature when at least one CNV gene is a member of this KEGG pathway. Finally, we incorporated in the classifier information regarding the gene expression variance from microarray expression experiments performed in 176 HapMap EBV cell lines, reasoning that dosage-sensitive genes tend to show less variable expression levels [36], [37].

#### Optimal balance among CNVs in the training set

**Figure 1:**
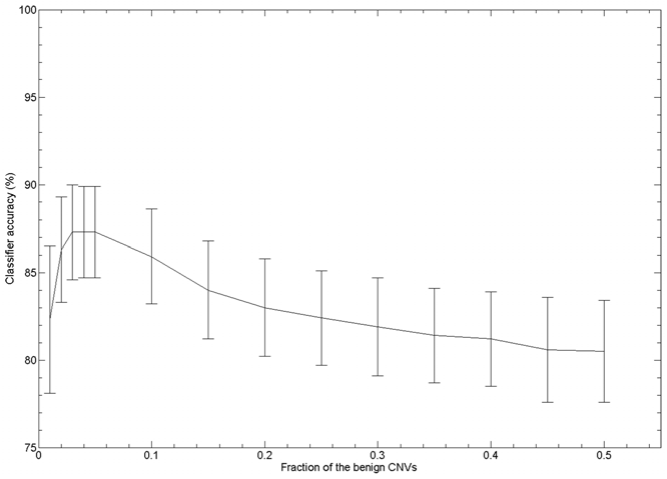
Effect of the imbalance between MR-associated and benign CNVs in the training set on the accuracy of the classifier. This figure shows the relationship between the fraction of available benign CNVs used in the training set and the accuracy of the classifier (calculated over 1,000 independent test and training runs). Maximum accuracy is achieved with a similar number of MR-associated and benign CNVs in the training set (∼5% of the benign CNV instances available).

The relative frequencies of the two different classes of CNV in the training set are very different (they are ‘imbalanced’). MR-associated CNVs are identified in ∼10% of MR patients screened and, for these, in the large majority of cases MR is attributable to only a single CNV (see **Introduction** for specific details regarding current clinical practise for identifying clinically-relevant CNVs). By contrast, 5 to 10 benign CNVs can be identified per non-patient individual, depending on the microarray platform being used [10]–[13]. We started by investigating the impact of this imbalance between the two CNV classes on the accuracy of the classifier during training. We performed 1,000 training and test runs of the classifier each with 30 different levels of imbalance between MR-associated and benign CNVs in the training set. Initially, a random selection was made consisting of half of all available benign CNVs (*n* = 1,413) and half of all MR-associated CNVs (*n* = 82). The remaining CNVs were used subsequently as test instances. The imbalance was then gradually decreased until equal numbers (*n* = 82) of MR-associated and benign CNVs were present in the training set (see **Materials and Methods**). The most imbalanced training set, consisting of 5.5% MR-associated and 94.5% benign CNVs (82∶1,413), produced a classifier with the lowest mean accuracy (80.4%±2.9%) (**Figure 1**). The highest mean accuracy (87.3%±2.6%) was achieved using a balanced training set containing 82 MR-associated and 82 benign CNVs: this scenario takes advantage of only 5% of all available benign CNVs for the training set.

#### Optimal selection of the training set

A consequence of using a balanced training set with equal numbers of MR-associated and benign CNVs is that not all available benign CNVs are used during training. In order to select the optimal training set we randomly re-sampled the training set over 10,000 iterations selecting 82 MR-associated CNVs and 82 benign CNVs, with the remaining benign CNVs being placed in the test set. A mean accuracy of 86% (±2.8%) was obtained from these iterations, which demonstrates that the classifier achieves a reasonable level of accuracy irrespective of which benign CNVs are selected for the training set. In addition, this analysis identified an optimal subset of CNVs for training which achieved a maximum accuracy of 95.7% and an area under the ROC curve of 0.98 when classifying the test set of CNVs. The resulting classifier using this optimal training set contains 5 tree nodes with univariate splits based on the CNV length, and on the segmental duplication, LINE, SINE and gene densities. The 6 leaves of the tree each contain a different Bayesian classifier based on all features used during training.

#### Feature Contribution to classification accuracy

**Figure 2:**
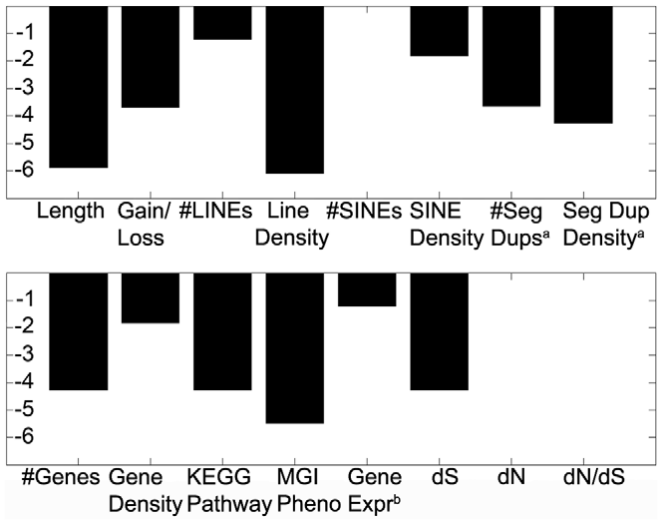
Analysis of the relative contribution of each genomic feature to the CNV classifier. Both structural and functional genomic features are evaluated for their impact on classification accuracy. This analysis is performed by measuring the decrease in accuracy of the classifier as each classification feature is removed individually. KEGG Pathway refers to the CNV region containing at least one gene implicated in a KEGG neurodegenerative pathway, and MGI Pheno refers to the CNV region containing at least one gene displaying a nervous system phenotype in a knockout mouse. Gene Expression refers to the stability of gene expression of genes present in the CNV. Removal of the LINE density from the classifier results in the largest decrease in accuracy (6%) whilst removing MGI knockout phenotypes results in a drop of 5% in accuracy. The number of SINE elements, the non-synonymous substitution rate (*d_N_*), and the ratio of the synonymous versus non-synonymous substitution rate (*d_N_*/*d_S_*) individually have no effect on the accuracy of the classifier.

The optimal training set was obtained by training the classifier on all 16 available features. To quantify the contribution of each feature to the accuracy of the classifier we used a leave-one-out policy for each feature, retrained the classifier and then measured the percentage decline in classification accuracy (**Figure 2**). However, in order to exclude the effect of length on the classifier, the features SINE, LINE, segmental duplication and gene count features were simultaneously removed with the length feature. For example, removing the LINE density or the length from the classifier resulted in a 6% decrease in accuracy, whilst removing the mouse MGI knock-out phenotypes resulted in more than a 5% decrease in accuracy. A 4.2% decrease in accuracy was measured when any one of the segmental duplication density, gene count, KEGG pathway or mean *d_s_* value was removed. Removing the CNV type (either gain or loss) resulted in a 3.7% decrease in accuracy. A similar decrease in accuracy was observed when removing the number of segmental duplications. Smaller effects were seen when any one of the LINE count, SINE density, gene density and gene expression features was removed from the classifier. By contrast, leaving out the number of SINE elements, mean *d_N_* value, or mean *d_N_*/*d_S_* ratio had little or no effect on the performance of the classifier. Consequently, these three features were excluded from the final classifier.

### Validation of the Classifier

#### Application to MR Syndromes

The Decipher database of known syndromes associated with genomic structural variants (https://decipher.sanger.ac.uk) provides a large set of pathogenic CNVs that is suitable for the independent validation of the classifier. In this database the genomic locations (based on microarray studies) of 58 syndromes are reported, 32 of which are associated with MR. We applied the classifier to these 32 genomic regions and found that 31 regions were classified as pathogenic. The 80kb critical region of Rubinstein-Taybi Syndrome was not correctly classified (**Table S1**). This region is a composite of overlapping microdeletions, ranging in size from 1.5–3.5Mb, identified in 3 individuals with this syndrome. When we tested these three regions individually each was classified as pathogenic. From this we concluded that the classifier was able to correctly identify known pathogenic CNVs.

#### Application to MR diagnostics

**Figure 3:**
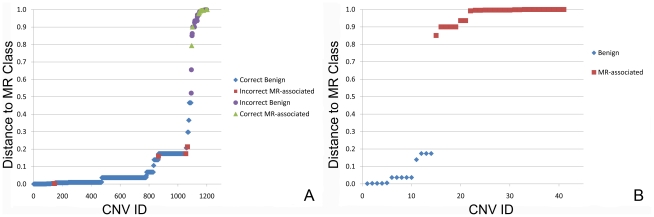
Benign CNVs are separable from MR-associated CNVs using a distance function that reflects the probability that a CNV belongs to the MR-associated CNV class. The CNVs are ranked and their probability of belonging to the MR-associated CNV class is plotted, A) 1,203 CNVs with known inheritance collected from routine diagnostics are classified with a sensitivity of 88% and a specificity of 94%. 1,085 of the 1,154 of the common inherited CNVs were correctly classified (blue), and 43 of 49 CNVs previously associated with MR were correctly classified as MR-associated (green). 6 CNVs which had been interpreted as not being associated with MR, were classified as MR-associated (red), as well as 69 CNVs classified as MR-associated which had previously been interpreted as benign (purple). B) Similarly, 41 rare inherited CNVs with unknown clinical significance are classified, 27 of which were classified as MR-associated with a MR distance >0.5 (green), and 14 were classified as benign (MR distance <0.5, blue).

We performed a second more extensive study to validate the accuracy of the classifier using an independent set of 584 MR patients in which 1,203 CNVs (the set “MR diagnostics”) had been identified during routine diagnostics using Affymetrix 250k SNP microarrays. These CNVs were identified as being associated with MR (*n* = 49) based on *de novo* occurrence and the absence of similar CNVs in the normal population, or as being benign CNVs (*n* = 1,154) known to be present in the normal population. Of the 1,203 CNVs in the validation set, 94% of the CNVs were classified correctly, with a sensitivity of 88% and a specificity of 94% (**Figure 3a**). More specifically, 43 of 49 MR-associated CNVs were correctly classified, 37 of which had a distance of less than 0.1 from the MR class, showing that these classifications have a high confidence. Each node of the NBTree contains a Bayesian classifier resulting in the most likely class (benign or MR-associated) for each CNV being predicted. In addition, the probability (a distance function) is calculated that a CNV belongs to the MR-associated CNV class or to the benign CNV class. The overall false positive rate was 0.05 and the false negative rate 0.12. The positive predictive value was 0.38 (indicating the number of CNVs correctly classified as MR, divided by the total number of CNVs classified as being MR). The negative predictive value was 0.99 (indicating the number of CNVs correctly classified as benign, divided by the total number of CNVs classified as benign) (**Table 2**). 1,085 of 1,154 benign CNVs (94%) were correctly classified whilst 69 (6%) were incorrectly classified as an MR-associated CNV (**Table 2**). To exclude the possibility that the initial training set did not contain sufficient biological coverage to represent the variance of each classification feature, and to train the optimal classifier, we combined the test and training sets and retrained the classifier. The accuracy of the resulting classifier was then tested on the validation set. The training set was jack-knifed to contain equal numbers of MR-associated and benign CNVs (*n* = 164) and 10,000 iterations were performed. The mean accuracy across all iterations was 76% and the maximum achieved accuracy was 94%, equal to that gained with the smaller training set. Thus we conclude that the training set with 82 MR-associated and 82 benign CNVs contained sufficient biological coverage to model the data accurately.

**Table 2:** Application of the classifier to CNVs obtained in routine diagnostics of patients with mental retardation.

Classifier Output	Validation Set (Rare de novo vs. commonly inherited CNVs)			Application Set (CNVs of unknown clinical significance)	
	MR CNVs (rare *de novo*)	Benign CNVs (commonly inherited)	Sub Total	Rare Inherited	Rare CNVs of unknown inheritance
**MR**	43	69	112	27	46
**Benign**	6	1,085	1,091	14	7
**Total**	49	1,154	1,203	41	53

The accuracy of the classifier developed was tested on an independent cohort of CNVs. Phase 1 contained the validation set of 1,203 CNVs known to be either rare *de novo* or commonly inherited. 43 of the 49 rare *de novo* CNVs known to be associated with MR were correctly classified, and 1,085 of the 1,154 common inherited CNVs known to be benign were correctly classified, thus giving an overall classification accuracy of 94%. The false positive rate was 0.05 and the false negative rate was 0.12. The positive predictive value was 0.38 and the negative predictive value was 0.99. Phase 2 consisted of the application set containing 94 CNVs of unknown clinical significance. Of the 41 rare inherited CNVs the classifier identified 27 CNVs as MR-associated and 14 as being benign. 53 candidate CNVs for which the inheritance could not be determined were also classified, from which 46 were classified as being MR and 7 CNVs were classified as being benign.

To further investigate the contribution of particular features to misclassification rates we calculated the mean values for each feature in the correctly and incorrectly classified CNV groups (**Table S2**). This highlighted some general differences between correctly and incorrectly classified CNVs. For example, 78% of the incorrectly classified benign CNVs were copy number gains and contained, on average, fewer segmental duplications than correctly classified benign CNVs. In addition, 86% of the correctly classified MR-associated CNVs contain at least one gene whose mouse orthologue knockout results in a nervous system phenotype, whereas only 33% of the incorrectly classified MR-associated CNVs contain such genes. We also noted that correctly classified MR-associated CNVs have an average genomic size of 7.7Mb, whereas CNVs incorrectly classified as benign have, on average, a much smaller size of 1.1Mb. Likewise, incorrectly classified benign CNVs also had a smaller average size (319kb) than benign CNVs correctly classified (492kb). We therefore investigated the accuracy of the classifier on 971 CNVs smaller than 1.1Mb in more detail. For these smaller CNVs, 9 of the 13 MR-associated CNVs as well as 890 of the 958 benign CNVs were classified correctly. The performance of the classifier on these small CNVs was comparable to the overall performance on the complete validation set with an accuracy of 93% and specificity of 93%, with the exception of sensitivity which dropped by 18% to 70%. Analysis of the genomic features in smaller CNVs showed that despite differences in CNV lengths, small MR-associated CNVs show many similarities to larger MR-associated CNVs such as similar SINE and gene densities (**Table S2**).

#### Application of the Classifier to CNVs of unknown clinical significance

Finally we sought to use our classifier on two further CNV datasets with unknown clinical significance, termed candidate CNVs and rare inherited CNVs. We first selected a set of 53 rare CNVs identified in the clinic, not known to vary in copy number among the general population, for which inheritance could not be established due to the unavailability of one or both parents. Due to their unknown inheritance and rare status, we are unable to determine using current diagnostic procedures whether these CNVs are indeed causal. In total, 46 of these 53 CNVs were classified as MR-associated CNVs (**Table 2**). We also applied the classifier to a set of rare, privately inherited CNVs that are not known to vary in the general population (**Figure 3b**). Twenty-seven of the 41 rare inherited CNVs were classified as an MR-associated CNV, and 14 were classified as a benign CNV (**Table 2**) displaying a significant enrichment in the number of CNVs classified as MR-associated when compared to size matched CNVs selected randomly from the genome (*p* = 7.0×10^−3^).

## Discussion

In this study we present a novel computational method to objectively identify clinically relevant CNVs using an NBTree classifier and 13 diverse genomic features. This is the first description of such a method applied to CNVs that can significantly improve interpretation of this important class of genomic variation. Our classification method has been validated on a set of 1,203 CNVs detected in 584 patients with MR, achieving a high accuracy (94%), with a sensitivity of 88% and a specificity of 94% (**Figure 3a**).

Several other computational methods have been developed previously to predict if disruption or disturbance of genomic elements have pathogenic consequences. Often these methods are focused on identifying disease genes or on predicting if mutation or splicing events are pathogenic [31]–[34]. Such methods make use of protein structure and stability measures, and phylogenetic or sequence conservation data [38], [39], and often cross-validate their predictions using OMIM (Online Mendelian Inheritance in Man) data [40]. These approaches may be less applicable for larger structural variants such as CNVs because they predict the effect of a single change on a single disease gene, rather than a large change involving many genes. Our approach differs in that we directly predict the causal CNV from genome-wide copy number scans on the basis of the distinguishing features of benign and disease-causing CNVs. In addition, OMIM does not provide a suitable source for validating the performance of a classification method for CNVs as dosage-sensitive genes are largely underrepresented in this database (<5% of the entries describe haploinsufficient genes [41]), and because a precise mapping of CNVs in OMIM is lacking. In contrast to OMIM, the Decipher database list of known syndromes (https://decipher.sanger.ac.uk) provides a suitable list of CNVs for external validation of the classifier with high-resolution mapping of their genomic locations. Our classification method correctly identified all the CNVs listed in this database as causing MR-associated syndromes.

The classifier incorporated specific knowledge about CNVs via 13 diverse structural and functional genomic features (including a number of different transposable element types). The proximity of these elements to CNVs has been reported previously and it has been hypothesized that they mediate the formation of recurrent CNVs [18], [26], [42]. We confirm previous results that benign CNVs are enriched in both LINE and segmental duplication elements [13], [28] and show that both the LINE density and the segmental duplication density substantially contribute to the classifier's accuracy **(Table S2)**. Previous studies have also reported that CNV gains are enriched in many of the same features as CNV losses [30]. Our feature contribution results support this finding: when the CNV type was removed from the classifier only a 3.7% decrease in accuracy was observed, and 7 additional features had a greater contribution to the classifier's accuracy. In addition to these transposable elements, we included functional genomic elements which have recently been shown to assist in distinguishing benign from MR-associated CNVs [30], [43]. The significant enrichment of MGI mouse nervous system phenotypes in MR loss CNVs has previously been reported [30]. We show that the MGI mouse knock-out phenotype feature is effective in distinguishing benign from MR-associated CNVs: 80% of all MR-associated CNVs contain one or more genes whose unique orthologue's disruption in mouse reveals a nervous system phenotype, whereas benign CNVs only rarely contain such genes **(Table S2)**.

Despite the MGI mouse phenotype dataset being incomplete, this feature contributes greatly to the classifier's accuracy (5%). To date, gene knockout experiments with recorded ontology based phenotype information have been performed for approximately 5,000 of the possible 15,287 genes with mouse 1∶1 orthologues [44], [45]. Furthermore the MGI phenotype data are included in the classifier as a binary feature (which is labelled as ‘true’; when a CNV contains 1 or more genes exhibiting a nervous system phenotype; MP:0003631). However, as the MGI phenotype dataset is incomplete, our approach is conservative with respect to missing values. This is because CNVs overlapping genes whose disruption does not result in a nervous system phenotype are weighted equally to those CNVs overlapping genes whose disruption phenotypes are currently unknown. Thus, we expect that increased coverage by the MGI mouse knock-out dataset will significantly improve the accuracy of the classifier. In addition, further genomic features such as CpG islands or conserved non-coding regions [46] can now be tested for their potential to improve the accuracy of this approach. Nevertheless, as the densities of many genomic features are strongly correlated [28], it is likely that the addition of further features to the classifier will not result in a substantial improvement in predictive power.

Most of the CNVs we used to train the classifier were identified on low-resolution (BAC–based) microarray platforms. In contrast, the replication set contained CNVs collected solely from Affymetrix 250k SNP microarrays. Despite the different microarray technologies used, only a negligible decrease in classification accuracy (−1.7%) was observed between the training and the replication set. This indicates that the classifier is platform-independent and will not require retraining when used on data generated from comparable microarray platforms.

MR-associated CNVs discovered thus far are, in general, larger than benign CNVs [30]. Previously developed CNV risk assessments for identifying disease-associated CNVs use a length greater than 3Mb as a distinguishing criterion [16]. Closer inspection of the MR-associated CNVs from our validation study indeed revealed a larger mean length (6.8Mb) compared to the benign CNVs (474kb). Despite this large size, 25% of the MR-associated CNVs in the validation set were smaller than 1.1Mb. We separately tested the accuracy of the classifier on CNVs smaller than 1.1Mb which revealed it to exhibit a decrease in sensitivity (−18%) but still a high accuracy (93%). As might be expected, small MR-associated CNVs showed a decrease in the number of MGI knock-out genes displaying a nervous system phenotype, but their SINE and gene densities are comparable to those of larger MR-associated CNVs **(Table S2)**. Importantly, the classifier was still able to correctly classify 9 of the 13 small MR-associated CNVs, demonstrating the advantage of the classifier in comparison to conventional interpretation methods which often are unable to clearly identify clinically relevant CNVs unless specific information about their genomic content is known [47].

Although current clinical interpretation of CNVs focuses on large, rare and *de novo* CNVs, an increasing number of genomic loci being reported show variable inheritance and penetrance [20]–[24]. Our replication study contained a number of such CNVs, including CNVs at 1q21.1 and 15q13.3 which, in addition, show variation in genomic size and content [20]–[23]. Three rare inherited CNVs encompassing the 1q21.1 critical region were all classified as associated with MR, even though their genomic breakpoints differed. Two rare *de novo* CNVs in the 15q13.3 region were classified differently, one as benign and one as pathogenic. In addition, three inherited CNVs at this locus were all classified as benign. Interestingly, the distal breakpoint for all five CNVs was identical whereas the proximal breakpoint of the four CNVs classified as benign was extended by an additional 150kb. This difference in classification is explained by the fact that the 150kb region showed a higher repeat element count and density due to repetitive elements surrounding the 15q13.3 critical region **(Table S2)** [23]. This particular example highlights the current challenge in clinical interpretation of CNVs which relies on the availability of large control datasets. We do not claim that our classification method replaces the need for such datasets. Our method does show that 27 out of 41 (66%) rare inherited CNVs identified in patients contain genomic features similar to previously recognized MR-associated CNVs, a significant proportion when compared to the remainder of the genome (**Figure 3b**). This provides independent support for the clinical relevance of this group of CNVs and shows that the interpretation of CNVs should not be limited to rare *de novo* CNVs with a fully penetrant dominant effect [48]. Furthermore, in the set of 53 rare CNVs with unknown inheritance, 46 CNVs were classified as being MR-associated, the vast majority with high confidence. These rare CNVs with unknown inheritance demonstrate strong similarities to rare *de novo* CNVs in that they have a low segmental duplication density, a high SINE density, often contain genes whose mouse knockouts result in nervous system phenotypes, have similar gene expression values and similar synonymous substitution rates. This suggests that these rare CNVs with unknown inheritance are indeed similar in pathoetiology to rare *de novo* CNVs and thus can be considered strong candidates for being causal CNVs. The ability of the classifier to identify such CNVs of unknown inheritance should be of great benefit to the diagnostic communities.

This CNV classifier may also be informative of disorders other than mental retardation. This is of particular relevance because CNVs have recently been associated with other neurodevelopmental disorders such as autism and schizophrenia [1], [4], [5] but screening for causal CNVs in these diseases has yet to be implemented in most clinics. Interestingly, many of the CNVs associated with autism and schizophrenia, as well as mental retardation, contain genes whose proteins are involved in neurotransmission or in synapse formation and maintenance. This supports the existence of shared biological pathways that are disrupted in each of these neurodevelopmental disorders [49]. Our CNV classifier trained on MR CNVs may therefore already have predictive power for CNVs in other neurological disorders. It is likely, however, that this predictive power can be further optimized by retraining the classifier using disease-specific CNVs. In addition, the KEGG and MGI features selected for the MR patient cohort are also easily configurable for pathways and phenotypes which are more relevant to these other disease cohorts. For this reason we have made the Java source code of the CNV classifier, called GECCO, freely available (see **Materials and Methods**).

In conclusion, we have developed a novel objective method to identify disease-associated CNVs which has overcome several limitations with current CNV interpretation methodology. Our NBTree classifier is able to distinguish between MR-associated CNVs and benign CNVs with high accuracy without the use of data from large control cohorts or parental samples. Results indicate that computational classification methods can be used for objectively prioritizing CNVs in clinical research and diagnostics. The tool for classifying CNVs, called GECCO (Genomic Classification of CNVs Objectively), as well as the Java source code, are readily available online. The benefits of such methods will increase with advancements in microarray technology, which already identifies many thousands of such structural variants per individual [50]–[53], and in whole genome resequencing technology,. Establishing objective criteria and methods for interpretation of these genomic variants will be crucial for implementation of these technologies in a clinical setting.

## Materials and Methods

### Classifier Development

In this study we investigate if rare *de novo* CNVs and commonly inherited CNVs could be successfully classified without the use of inheritance information. In order to achieve this we collected from the literature a large number of rare CNVs known to be *de novo* (*n* = 164) and a number of common CNVs known to be benign (*n* = 1,413). These CNVs were used for training and testing the classifier. A total of 20 genomic features were initially investigated. Initially 16 features were selected as attributes during the development of the classifier, which was then further optimized to a set of 13 features **(** **Table 1** **)**. To test the accuracy of the classifier we first tested the classifier on a set of CNVs previously identified as being associated with MR (Decipher known syndromes), and then created an independent validation set containing rare *de novo* and common inherited CNVs, collected from routine diagnostics, to be used in a validation study (MR diagnostic CNVs). Finally two application sets were created containing CNVs without a clinical interpretation that were either a) candidate CNVs, due to unavailability of parental samples, or b) rare privately inherited CNVs.

### Data Sets

The CNVs used during the training and test phase (164 rare *de novo* CNVs termed “MR-associated CNVs” and 1,413 common inherited CNVs termed “benign CNVs”) were identified on a number of different microarray platforms in previously published studies [15], [19], [28], [30]. All aberrations were mapped using HG17 coordinates and converted when necessary using UCSC liftOver [54]. The Decipher known syndromes' (https://decipher.sanger.ac.uk/) dataset contained 32 pathogenic CNVs based on microarray studies and associated with MR. The remaining 26 syndromes were excluded as they do not have either mental retardation as a prominent phenotype or a fully penetrant phenotype.

MR Diagnostics and application datasets were collected through in-house routine diagnostics using Affymetrix 250k SNP microarrays (Affymetrix, Santa Clara, USA), and consisted of 584 samples containing 1,297 CNVs. Regions were excluded that contained fewer than 5 microarray targets, that were smaller than 10kb in size or that were the result of a mosaic or complex chromosomal aberration. In total, the validation/application set contained 49 rare *de novo* CNVs, 41 rare inherited CNVs, 53 candidate CNVs and 1,154 common inherited CNVs.

### Classifier Training

Initially a training set was created by randomly selecting 82 of the 164 rare *de novo* CNVs with an equal number of commonly inherited CNVs. The remaining CNVs were placed in the test set. The NBTree classification algorithm as implemented in Weka 3.6.0 [55] was selected and incorporated into our Java based tool called GECCO (Genomic Classification of CNVs Objectively). An executable version and all source code for GECCO are readily available via http://genomegecco.sourceforge.net. NBTree is a hybrid method combining a decision tree with Naïve-Bayesian classifiers. The Naïve-Bayesian classifiers calculate the posterior probability (a distance function) that the CNV belongs to either class (MR-associated CNV or benign CNV).The definition of the training set was then investigated. Given the imbalance that exists in the data (see Results) we sought to incorporate this prior into the training set. We tested increasingly imbalanced versions of the training set, starting with the most unbalanced training set, by placing half of all available CNVs in the training set (164 *de novo* and 2826 common inherited), and gradually decreasing the imbalance until the training set contained only 5% (*n* = 143) of all available common inherited CNVs. The training set imbalance was then further tested in 1% decrements until the minimum was reached of 82 rare *de novo* CNVs and 28 common inherited CNVs. Once an optimal balance of CNV classes in the training set was identified the optimal subset of the CNVs in the training set was determined. This was achieved by randomly selecting CNVs as training and test instances over 10,000 iterations and then identifying the set that produced the maximum accuracy. In addition, enrichment analysis of the rare inherited CNVs was performed by generating 1,000 sets of random genomic regions matched for size against the rare inherited CNVs and the proportion of sets with greater than or equal to 27 CNVs classified as being MR was calculated.

### Genomic Features used for Classification

In total 20 different genomic features were investigated as potential classifier attributes. The variance inflation factor (VIF) was used to measure the co-linearity within the model across the repeat, gene and evolution measures (simple repeats, repeat masker, LINE, SINE, long terminal repeats, RNA gene elements, segmental duplications, ENSEMBL genes, mean non-synonymous substitution rate (*d_N_*), synonymous substitution rate (*d_S_*) and the *d_N_*/*d_S_* ratio of genes). Based on the VIF, features were removed until the model contained only independent features resulting in 16 different structural and functional genomic features that were used subsequently for training the classifier **(** **Table 1** **)**. The included structural features were CNV type (loss∶gain), CNV length, the numbers of LINE, SINE and segmental duplication elements lying within the CNV, as well as the densities of the LINE, SINE and segmental duplication elements. The density values were determined as the number of elements per base pair. Segmental duplications were downloaded from the UCSC table genomicSuperDups. The numbers of LINE and SINE elements were extracted from the UCSC table from rmsk and the RNA gene elements from sno/miRNA.

The functional genomic features consisted of the gene count, gene density and the variance in gene expression levels, the mean non-synonymous substitution rate (*d_N_*), synonymous substitution rate (*d_S_*) and the *d_N_*/*d_S_* ratio. In addition KEGG pathway and MGI knockout phenotypes were added as features. Genes involved in the KEGG (Kyoto Encyclopedia of Genes and Genomes) neurodegenerative pathway (hsa01510) [56] were added as a categorical feature. This pathway includes KEGG genes belonging to KEGG Pathways section 5.2, namely Alzheimer's disease (KEGG pathway 05010), Parkinson's disease (KEGG pathway 05020), Amyotrophic Lateral Sclerosis (KEGG pathway 05030), Huntington's disease (KEGG pathway 05040), Dentatorubropallidoluysian atrophy (KEGG pathway 05050) and Prion Diseases (KEGG pathway 05060). KEGG genes were mapped to NCBI Entrez genes using associations provided by KEGG. Genes which were annotated as having the MGI mouse knockout phenotype, MP:0003631: nervous system phenotype were also added as a categorical feature. These genes were identified via human NCBI genes whose mouse orthologue's disruption had been assayed and were obtained from the Mouse Genome Informatics (MGI) resource (http://www.informatics.jax.org, version 3.54) [45]. Substitution rates were obtained from EPGD [57]. The stable expression was calculated via the standard deviation of log_2_ intensities across 176 Hapmap cell lines (CEU and YRI) hybridized onto an Affymetrix GeneChip Human Exon 1.0 ST array (GSE7761).

## Supporting Information

Figure S1 Workflow used to develop the classifier. The classifier is able to distinguish between MR CNVs and benign CNVs based upon solely genomic features without the use of inheritance information. Several classification methods are tested. A training set consisting of both MR and benign CNVs is selected and the genomic features extracted. These data are used to train the classifier which is then evaluated with a separate test set of CNVs. The process of training set selection is repeated until an optimal performance is obtained. Subsequently, the classifier is validated on an independent set of MR and benign CNVs.
(0.05 MB DOC)

Table S1 Classification Results of 32 MR syndromes from the DECIPHER database. The chromosome location, syndrome name, as well as the CNV length and type are given. The classification results are shown with the MR distance measure, showing the confidence of each classification decision.
(0.06 MB DOC)

Table S2 Mean (and standard deviation) of each genomic feature used by the classifier during the validation and application studies. For each class of CNV the feature mean and (standard deviation) for the correctly and incorrectly classified CNVs are indicated.
(0.06 MB DOC)
