# Supplementary material for: Accurate Distinction of Pathogenic from Benign CNVs in Mental Retardation
Source: PLoS Comput Biol. 2010 Apr 22;6(4):e1000752. doi: 10.1371/journal.pcbi.1000752 (PMC2858682; doi:10.1371/journal.pcbi.1000752)
Supplement: Figure S1 — Workflow used to develop the classifier. The classifier is able to distinguish between MR CNVs and benign CNVs based upon solely genomic features without the use of inheritance information. Several classification methods are tested. A training set consisting of both MR and benign CNVs is selected and the genomic features extracted. These data are used to train the classifier which is then evaluated with a separate test set of CNVs. The process of training set selection is repeated until an optimal performance is obtained. Subsequently, the classifier is validated on an independent set of MR and benign CNVs. (0.05 MB DOC) [file pcbi.1000752.s001.doc]

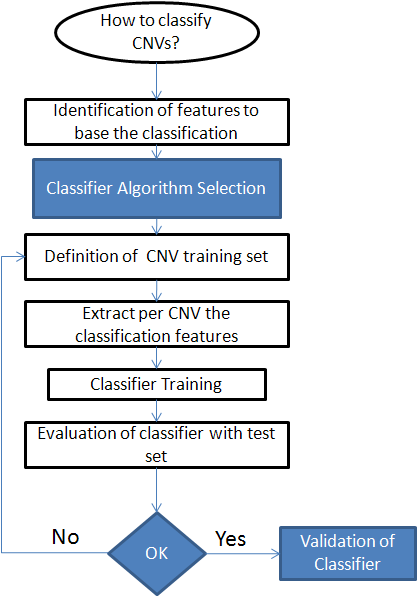


Supplementary Figure 1: Workflow used to develop the classifier. The classifier is able to distinguish between MR CNVs and benign CNVs based upon solely genomic features without the use of inheritance information. Several classification methods are tested. A training set consisting of both MR and benign CNVs is selected and the genomic features extracted. These data are used to train the classifier which is then evaluated with a separate test set of CNVs. The process of training set selection is repeated until an optimal performance is obtained. Subsequently, the classifier is validated on an independent set of MR and benign CNVs.
