# Supplementary material for: Accurate Distinction of Pathogenic from Benign CNVs in Mental Retardation
Source: PLoS Comput Biol. 2010 Apr 22;6(4):e1000752. doi: 10.1371/journal.pcbi.1000752 (PMC2858682; doi:10.1371/journal.pcbi.1000752)
Supplement: Table S1 — Classification Results of 32 MR syndromes from the DECIPHER database. The chromosome location, syndrome name, as well as the CNV length and type are given. The classification results are shown with the MR distance measure, showing the confidence of each classification decision. (0.06 MB DOC) [file pcbi.1000752.s002.doc]

**Supplementary Table 1:** Classification Results of 32 MR syndromes from the DECIPHER database. The chromosome location, syndrome name, as well as the CNV length and type are given. The classification results are shown with the MR distance measure, showing the confidence of each classification decision.

| ID | ChrNr | Syndrome | Length (kb) | Sort | Classifier Distance to MR Class | Classified as MR |
| --- | --- | --- | --- | --- | --- | --- |
| 1 | chr1 | 1p36 microdeletion syndrome | 5,241 | Loss | 0.996 | Yes |
| 2 | chr2 | 2p15-16.1 microdeletion syndrome | 3,997 | Loss | 0.996 | Yes |
| 3 | chr2 | 2q33.1 deletion syndrome | 8,169 | Loss | 0.977 | Yes |
| 4 | chr2 | 2q37 monosomy | 2,951 | Loss | 0.996 | Yes |
| 5 | chr3 | 3q29 microdup | 1,826 | Gain | 0.987 | Yes |
| 6 | chr3 | 3q29 microdeletion syndrome | 1,665 | Loss | 0.992 | Yes |
| 7 | chr4 | Wolf-Hirschhorn syndrome | 1,885 | Loss | 0.992 | Yes |
| 8 | chr5 | Cri du Chat syndrome | 11,564 | Loss | 0.996 | Yes |
| 9 | chr5 | Sotos syndrome | 2,186 | Loss | 0.992 | Yes |
| 10 | chr7 | Williams-Beuren syndrome | 2,095 | Loss | 0.992 | Yes |
| 11 | chr8 | 8p23.1 deletion syndrome | 3,473 | Loss | 0.996 | Yes |
| 12 | chr9 | 9q subtelemeric syndrome | 750 | Loss | 0.793 | Yes |
| 13 | chr11 | Potocki-Shafter syndrome | 2,025 | Loss | 0.992 | Yes |
| 14 | chr12 | 12q14 microdeletion syndrome | 3,574 | Loss | 0.996 | Yes |
| 15 | chr15 | Prader-Willi syndrome | 3,949 | Loss | 0.996 | Yes |
| 16 | chr15 | Angelman Type1 syndrome | 5,803 | Loss | 0.996 | Yes |
| 17 | chr15 | Angelman Type2 syndrome | 4,921 | Gain | 0.987 | Yes |
| 18 | chr15 | 15q26 overgrowth | 3,163 | Gain | 0.987 | Yes |
| 19 | chr15 | 15q13.3 microdeletion syndrome | 1,931 | Loss | 0.992 | Yes |
| 20 | chr15 | 15q24 recurrent microdeletion syndrome | 1,785 | Loss | 0.992 | Yes |
| 21 | chr16 | Rubinstein-Taybi syndrome | 80 | Loss | 0.012 | No |
| 22 | chr16 | 16p11.2-12.2 microdeletion syndrome | 7,428 | Loss | 0.996 | Yes |
| 23 | chr17 | Miller-Dieker syndrome | 2,344 | Loss | 0.992 | Yes |
| 24 | chr17 | Smith-Magenis syndrome | 3,703 | Loss | 0.985 | Yes |
| 25 | chr17 | NF1-microdeletion syndrome | 999 | Loss | 0.992 | Yes |
| 26 | chr17 | 17q21.3 microdeletion syndrome | 511 | Loss | 0.647 | Yes |
| 27 | chr22 | 22q11 duplication syndrome | 2,555 | Gain | 0.985 | Yes |
| 28 | chr22 | 22q11 deletion syndrome (Di George) | 3,640 | Loss | 0.996 | Yes |
| 29 | chr22 | 22q13 deletion syndrome (Phelan-Mcdermid) | 101 | Loss | 0.97 | Yes |
| 30 | chr22 | 22q11.2 distal del | 1,580 | Loss | 0.996 | Yes |
| 31 | chrX | Pelizaeus-Merzbacher syndrome | 490 | Gain | 0.692 | Yes |
| 32 | chrX | xq28 (MECP2) duplication | 377 | Gain | 0.9 | Yes |
