# Supplementary material for: Accurate Distinction of Pathogenic from Benign CNVs in Mental Retardation
Source: PLoS Comput Biol. 2010 Apr 22;6(4):e1000752. doi: 10.1371/journal.pcbi.1000752 (PMC2858682; doi:10.1371/journal.pcbi.1000752)
Supplement: Table S2 — Mean (and standard deviation) of each genomic feature used by the classifier during the validation and application studies. For each class of CNV the feature mean and (standard deviation) for the correctly and incorrectly classified CNVs are indicated. (0.06 MB DOC) [file pcbi.1000752.s003.doc]

**Supplementary** **Table 2:** Mean (and standard deviation) of each genomic feature used by the classifier during the validation and application studies. For each class of CNV the feature mean and (standard deviation) for the correctly and incorrectly classified CNVs are indicated.

|  | **n^1^** | **Length (bp)** | **Sort (%Gain)** | **# LINE** | **LINE Density (bp)** | **SINE Density (bp)** | **# Seg Dup^2^** | **SegDup Density (bp)** | **# Genes** | **Gene Density (bp)** | **KEGG Pathway^3^** | **MGI Phenotypes^4^** | **Genes Expression^5^** | **Evolution** *d_S_***^6^** |
| --- | --- | --- | --- | --- | --- | --- | --- | --- | --- | --- | --- | --- | --- | --- |
| **MR CNVs** | 49 | 6,868,119 (13,483,761) | 0.143 | 3,413 (7,621) | 0.00045 (0.0001) | 0.00076 (0.0004) | 112 (218) | 0.00003 (0.00004) | 65 (111) | 0.00002 (0.00002) | 0.061 | 0.796 | 0.157 (0.06) | 0.04  (0.06) |
| **Rare Inherited CNVs** | 41 | 1,179,475  (1,998,736) | 0.634 | 545 (820) | 0.00048 (0.00010) | 0.00055 (0.00031) | 13 (24) | 0.00001 (0.00002) | 7 (13) | 0.00001 (0.00001) | 0.000 | 0.171 | 0.178 (0.117) | 0.003 (0.006) |
| **Benign CNVs** | 1,154 | 482,023 (582,532) | 0.677 | 227  (274) | 0.00047 (0.00010) | 0.00059 (0.00039) | 73 (117) | 0.00008 (0.00008) | 3 (3) | 0.00001 (0.00002) | 0.001 | 0.000 | 0.121 (0.137) | 0.006 (0.034) |
| **Candidate CNVs** | 53 | 2,567,026 (3,926,881) | 0.547 | 1,246 (2,028) | 0.00047 (0.00008) | 0.00069 (0.00032) | 56 (115) | 0.00002 (0.00003) | 34 (50) | 0.00001 (0.00002) | 0.000 | 0.509 | 0.151 (0.088) | 0.014 (0.073) |
|  |  |  |  |  |  |  |  |  |  |  |  |  |  |  |
| **Correctly Predicted MR CNVs** | 43 | 7,676,020 (14,221,898) | 0.140 | 3,816 (8,062) | 0.00045 (0.0001) | 0.00078 (0.0004) | 115 (226) | 0.00002 (0.00003) | 72 (117) | 0.00002 (0.00002) | 0.070 | 0.860 | 0.159 (0.06) | 0.009 (0.016) |
| **Incorrectly Predicted MR CNVs** | 6 | 1,078,163 (725,264) | 0.167 | 524 (384) | 0.00048 (0.0001) | 0.00063 (0.0002) | 92 (159) | 0.00006 (0.00007) | 15 (17) | 0.00001 (0.00001) | 0.000 | 0.333 | 0.144 (0.032) | 0.000 (0.000) |
|  |  |  |  |  |  |  |  |  |  |  |  |  |  |  |
| **Correctly Predicted Benign CNVs** | 1,085 | 492,408 (596,653) | 0.670 | 232 (280) | 0.00047 (0.00010) | 0.00058 (0.00039) | 77 (119) | 0.00008 (0.00008) | 3 (3) | 0.00001 (0.00002) | 0.000 | 0.006 | 0.120 (0.139) | 0.006 (0.031) |
| **Incorrectly Predicted Benign CNVs** | 69 | 318,731 (223,884) | 0.783 | 149 (112) | 0.00046 (0.0001) | 0.00073 (0.00044) | 8 (20) | 0.00002 (0.00003) | 3 (5) | 0.00001 (0.00002) | 0.000 | 0.174 | 0.160 (0.081) | 0.016 (0.082) |
| **Small MR CNVs** | 13 | 659,115 (336,878) | 0.231 | 277 (155) | 0.00042 (0.00006) | 0.00084 (0.00049 | 10 (17) | 0.00001 (0.00002) | 13 (14) | 0.00002 (0.00003) | 0 | 0.538 | 0.164 (0.072) | 0.009 (0.024) |

^1^n=Number of CNVs; ^2^#Sep Dup = Number of Segmental Duplications; ^3^KEGG Path=The percentage of CNVs whose overlapping genes are contained in hsa01510; ^4^MGI Phenotypes=The percentage of CNVs whose overlapping genes are reported to have the phenotype MP:0003631; ^5^Gene Expression = mean standard deviation of log_2_ intensities across 176 Hapmap cell lines; ^6^Synonymous substitution rate
